# Efficacy of SGLT2 Inhibitors on Clinical Outcomes After Transcatheter Aortic Valve Replacement: A Systematic Review and Meta‐Analysis

**DOI:** 10.1002/edm2.70184

**Published:** 2026-02-26

**Authors:** Shaikh Muhammad Daniyal, Naveen Murad Khatoon, Anas Rasool, Isbah Gul, Sabula Tabish, Shireen Asifa, Ayan Khalid, Zunaira Aftab, Muhammad Burhan, Syeda Laiba Fahim, Habiba Tauqir Gondal, Muhammad Asfandyar Nadir, Syed Zaeem Ahmed, Danish Ali Ashraf, Somaiya Ahmed

**Affiliations:** ^1^ Department of Medicine Dow University of Health Sciences Karachi Pakistan; ^2^ Department of Medicine Allama Iqbal Medical College Lahore Pakistan; ^3^ Department of Medicine TruGift Health LLC Wilmington Delaware USA; ^4^ Department of Medicine Sir Salimullah Medical College Dhaka Bangladesh

**Keywords:** aortic stenosis, heart failure, meta‐analysis, sodium‐glucose cotransporter 2 inhibitors, transcatheter aortic valve replacement

## Abstract

**Background:**

Sodium‐glucose cotransporter 2 (SGLT2) inhibitors improve cardiovascular (CV) outcomes in patients with heart failure (HF) and type 2 diabetes. Their effect in patients undergoing transcatheter aortic valve replacement (TAVR) for severe aortic stenosis (AS), however, remains unclear. This study evaluated whether SGLT2 inhibitors reduce all‐cause mortality and HF hospitalisations after TAVR.

**Methods:**

A systematic search of PubMed, ScienceDirect, Cochrane (CENTRAL), Scopus and Embase was performed through April 2025 for studies comparing post‐TAVR outcomes between SGLT2 inhibitor users and non‐users. Outcomes of interest included a composite of all‐cause mortality or heart failure (HF) hospitalisation, along with the individual components of all‐cause mortality and HF hospitalisation. All outcomes were extracted at 1 year. Pooled hazard ratios (HRs) with 95% confidence intervals (CIs) were calculated using a random‐effects model. Heterogeneity was quantified using *I* ^2^ statistics. Analyses were conducted in R (version 4.4.2).

**Results:**

Three studies (1 RCT, 2 Observational) comprising 3187 patients met inclusion criteria. SGLT2 inhibitor use was associated with a reduced risk of the composite outcome (HR: 0.75; 95% CI: 0.65, 0.86; *p* < 0.01). Individually, therapy lowered all‐cause mortality (HR: 0.72; 95% CI: 0.53, 0.96; *p* = 0.03) and HF hospitalisations (HR: 0.74; 95% CI: 0.61, 0.90; *p* < 0.01).

**Conclusion:**

In patients with severe AS undergoing TAVR, SGLT2 inhibitors were associated with significant reductions in all‐cause mortality and HF hospitalisations. These findings suggest a promising role for SGLT2 inhibitors in improving post‐TAVR outcomes. However, given the limited data, larger randomised clinical trials are necessary to consolidate these findings.

**Trial Registration:** CRD420251132729

## Graphical abstract

SGLT2 inhibitor therapy was associated with a significantly lower risk of the composite outcome of all‐cause mortality or heart failure hospitalization at one year, as well as each component individually.

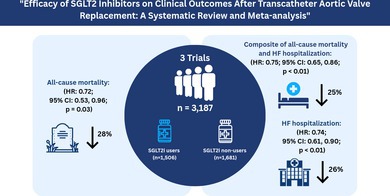

**Abbreviations**
AKI: acute kidney injury
AS: aortic stenosis
CV: cardiovascular
EACTS: European Association for Cardio Thoracic Surgery
ESC: Current European Society of Cardiology
HF: heart failure
HFpEF: heart failure with preserved ejection fraction
LVEF: left ventricular ejection fraction
NOS: Newcastle‐Ottawa Scale
NT‐proBNP: N‐terminal pro‐B‐type natriuretic peptide
PRISMA: Preferred Reporting Items for Systematic Reviews and Meta‐Analyses
RCT: randomisedrandomized controlled trial
RoB: risk of bias
RoB 2: Revised Cochrane Risk‐of‐Bias Tool
SGLT2: sodium‐glucose cotransporter 2
TAVR: transcatheter aortic valve replacement

## 1 Introduction

Aortic stenosis (AS) represents the most prevalent form of valvular heart disease, contributing to approximately 43% of all valvular conditions [1]. The global burden of AS has risen substantially, driven largely by an aging population [2]. Transcatheter aortic valve replacement (TAVR) has emerged as an established standard of care for AS, particularly for older adults or those at high surgical risk [3]. Despite success, subsequent morbidity and mortality remain substantial in this population, with heart failure (HF) readmissions posing a major challenge to long‐term outcomes [4, 5].

Sodium‐glucose cotransporter 2 (SGLT2) inhibitors, originally developed for diabetes, have been proven to reduce HF hospitalisations and cardiovascular (CV) mortality in patients with type 2 diabetes, HF, or chronic kidney disease [6]. However, major randomised controlled trials (RCTs) of SGLT2 inhibitors in HF with preserved ejection fraction (HFpEF) – including EMPEROR‐Preserved, SOLOIST‐WHF and DELIVER – excluded patients with significant valvular disease [7, 8, 9]. Notably, the ability of SGLT2 inhibitors to reverse cardiac remodelling supports their potential therapeutic role in AS, where chronic pressure overload leads to remodelling [10, 11].

Current European Society of Cardiology (ESC) and European Association for Cardio‐Thoracic Surgery (EACTS) guidelines recommend TAVR for patients with symptomatic severe AS who are ≥ 70 years of age, provided they have suitable anatomy and transfemoral access [12]. However, because AS has traditionally been viewed as a mechanical obstruction rather than a metabolic disease, post‐TAVR pharmacological therapies have received limited attention, leaving a noteworthy gap in current clinical guidelines [13]. Addressing this gap, we synthesised evidence from high‐quality clinical trials to evaluate the effect of SGLT2 inhibitor therapy in patients with severe AS undergoing TAVR.

## 2 Methods

This systematic review and meta‐analysis was conducted in adherence to the Preferred Reporting Items for Systematic Reviews and Meta‐Analyses (PRISMA) guidelines [14], and the review protocol was registered with PROSPERO (CRD420251132729).

### 2.1 Literature Search

An extensive literature search of PubMed, ScienceDirect, Cochrane (CENTRAL), Scopus and Embase was performed to identify articles evaluating the use of SGLT2 inhibitors in patients undergoing TAVR. The search was conducted from inception up until April 30, 2025. Additionally, no extra filters were used during the search, and a manual literature search was also performed. The search strategy included relevant PubMed entry terms and Medical Subject Headings (MeSH) terms, including (‘Transcatheter Aortic Valve Replacement’ OR TAVR OR ‘Transcatheter Aortic Valve Implantation’ OR TAVI OR ‘Catheter‐Based Aortic Valve Replacement’ OR ‘Percutaneous Aortic Valve Replacement’ OR ‘Percutaneous Aortic Valve Implantation’ OR ‘Transcatheter Aortic Valve Procedure’ OR ‘Transfemoral Aortic Valve Replacement’ OR ‘Transapical Aortic Valve Replacement’ OR ‘Aortic Valve Implantation’ OR ‘Minimally Invasive Aortic Valve Replacement’) AND (‘SGLT2 inhibitors’ OR ‘SGLT‐2 inhibitors’ OR ‘Sodium‐Glucose Cotransporter 2 Inhibitors’ OR ‘Sodium‐Glucose Co‐Transporter 2 Inhibitors’ OR ‘Sodium‐Glucose Transporter 2 Inhibitors’ OR ‘Glucose Cotransporter Inhibitors’ OR ‘SGLT2 blockade’ OR ‘SGLT2 inhibition’ OR ‘Sodium‐glucose transporter inhibitors’ OR ‘SGLT inhibitor’ OR ‘Gliflozin’ OR ‘Dapagliflozin’ OR ‘Empagliflozin’ OR ‘Canagliflozin’ OR ‘Ertugliflozin’ OR ‘Ipragliflozin’ OR ‘Remogliflozin etabonate’) AND (Aortic Valve Stenosis [Mesh] OR Aortic stenosis OR Severe Aortic Stenosis OR ‘Symptomatic Aortic Stenosis’ OR ‘Degenerative Aortic Stenosis’ OR ‘Calcific Aortic Stenosis’ OR ‘Senile Aortic Stenosis’ OR ‘Aortic Valve Narrowing’ OR ‘Aortic Valve Obstruction’ OR ‘Left Ventricular Outflow Obstruction’ OR ‘Aortic Valve Disease’). The detailed search strategy used in each database is presented in Table S1.

### 2.2 Study Selection Criteria

All identified citations were imported into Rayyan.ai for deduplication [15]. Two reviewers (A.R and A.K) blindly and independently screened titles and abstracts by using predetermined inclusion and exclusion criteria. Full texts of potentially eligible studies were retrieved and assessed for final inclusion. Any disagreements between the two reviewers were resolved through discussion with a third reviewer (N.M.K) until a consensus was reached. Studies were included if they met the following criteria: (1) Enrolled adult patients undergoing TAVR who were prescribed SGLT2 inhibitors. (2) Reported on at least one of the pre‐specified clinical outcomes of interest: (a) a composite of all‐cause mortality and HF hospitalisation, (b) all‐cause mortality and (c) HF hospitalisation (3) Were designed as RCTs or observational studies. (4) Were published in the English language. Studies were excluded for the following reasons: (1) They were case reports, case series, editorials, or review articles. (2) They involved non‐human subjects or paediatric populations. (3) They did not report any of the pre‐specified outcomes of interest. (4) They were published in a language other than English.

### 2.3 Data Extraction and Quality Assessment

Two reviewers (N.M.K. and I.G.) independently extracted data on baseline characteristics and adjusted effect estimates for all clinical outcomes using a standardised Microsoft Excel spreadsheet. Any discrepancies were resolved through cross‐checking.

Quality assessment was carried out independently by two reviewers (S.L.F and H.T.G). Any discrepancies were resolved through discussion until a consensus was reached. Risk of bias in RCTs was assessed using the Revised Cochrane Risk‐of‐Bias Tool (RoB 2), evaluating five domains categorised as low risk, some concerns, or high risk [16]. For observational studies, the Newcastle‐Ottawa Scale (NOS) was used, covering selection, comparability and outcome. Scores of 7–9 indicated low risk, 4–6 moderate risk and 0–3 high risk [17].

### 2.4 Statistical Analysis

Statistical analyses were performed using R software (version 4.4.2). For dichotomous time‐to‐event outcomes, Hazard Ratios (HRs) with 95% Confidence Intervals (CIs) were extracted from the included studies. In accordance with standard meta‐analytic practice for time‐to‐event data, these values were converted to the log HR and its variance for pooling.

To ensure comparability, outcome data at the 1‐year time point were used for all pooled analyses. A random‐effects model was applied, using the restricted maximum likelihood (REML) estimator to calculate the between‐study variance [18]. Results were presented graphically using forest plots. Statistical heterogeneity was quantified with the *I* ^2^ statistic, with values > 50% considered to represent substantial heterogeneity. We conducted a leave‐one‐out sensitivity analysis, sequentially excluding each study to assess its individual impact on the pooled estimate. Furthermore, subgroup analysis was performed by study design: RCT vs. observational. Assessment of publication bias was not performed due to the limited number of studies (*n* = 3) included in each analysis.

## 3 Results

**FIGURE 1:**
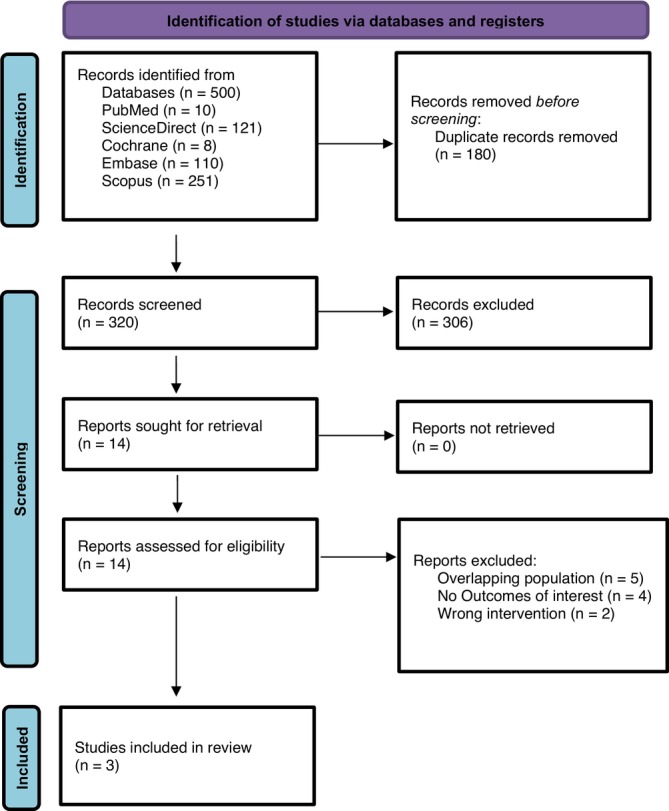
PRISMA flowchart showing the screening and study selection process.

A comprehensive search across five major databases identified a total of 500 records. After removing 180 duplicate entries, 320 records were available for title and abstract screening. Of these, 306 were excluded for not meeting the eligibility criteria. 14 full‐text articles were sought for retrieval. Following detailed assessment, 11 were excluded. Ultimately, three studies met all predefined inclusion criteria and were included in the final analysis. The PRISMA flow diagram summarises the study identification, screening and selection process (Figure 1).

### 3.1 Study Characteristics

A total of 3 studies were included, comprising 3187 patients undergoing TAVR with or without SGLT2 inhibitors [19, 20, 21]. Furthermore, the included studies were published between 2024 and 2025, with one observational study based in the United States (Thakkar et al.), one observational multicentre study in Europe (Paolisso et al.) and one RCT (Roubin et al.). Sample size ranged from 311 to 1654 patients. The baseline characteristics of the included studies are summarised in Table 1.

**TABLE 1:** Baseline characteristics of the included studies.

Author, year	Roubin et al. 2025	Paolisso et al. 2024	Thakkar et al. 2024
Study design	Randomised Controlled Trial	Retrospective cohort	Retrospective Cohort
Intervention	Oral dapagliflozin and standard care	On SGLT2 inhibitors therapy	On SGLT2 inhibitors therapy
Control	Standard care alone	Not on SGLT2 inhibitors therapy	Not on SGLT2 inhibitors therapy
Follow‐up	1 year	2 years	1 year
Number of patients (total)	1222	311	1654
Number of patients (Intervention/Control)	605/617	74/237	827/827
Female sex % (Intervention/Control)	49.4/49.4	21.6/36.7	N/A
Age, years (mean ± SD) (Intervention/Control)	82.4 ± 5.6/82.4 ± 5.5	77/81	N/A
**Cardiovascular disease history and risk factors: *n* (%) (Intervention/control)**			
T2DM	264 (43.6)/273 (44.2)	74 (100)/237 (100)	N/A
Hypertension	518 (85.6)/519 (84.0)	58 (78.4)/203 (85.7)	N/A
CAD	237 (39.2)/197 (31.9)	53 (71.6)/146 (61.6)	N/A
Afib	250 (41.3)/274 (44.3)	41 (55.4)/95 (40.1)	N/A
All‐cause mortality: *n* (%) (Intervention/Control)	47 (7.8)/55 (8.9)	7 (9.5)/92 (38.8)	N/A
HF Hospitalisations: *n* (%) (Intervention/Control)	45 (7.4)/66 (10.7)	7 (9.5)/59 (24.9)	N/A

Abbreviations: ACE, angiotensin‐converting enzyme; Afib, atrial fibrillation; ARB, angiotensin receptor blocker; CABG, coronary artery bypass grafting; CAD, coronary artery disease; CKD, chronic kidney disease; COPD, chronic obstructive pulmonary disease; HF, heart failure; MV surgery, mitral valve surgery; N/A, data not available; PCI, percutaneous coronary intervention; SAVR, surgical aortic valve replacement; SGLT2 inhibitors, sodium–glucose co‐transporter 2 inhibitors; T2DM, type 2 diabetes mellitus.

*Population (P)*: Included studies evaluated patients with severe AS undergoing TAVR, with comorbidity‐specific inclusion criteria. Paolisso et al. enrolled consecutive patients with type 2 diabetes mellitus, LVEF < 50% and extravalvular cardiac damage, excluding those with severe renal dysfunction, active malignancy, inadequate echocardiographic data, or follow‐up < 6 months. Roubin et al. included patients with prior AS‐related HF and at least one high‐risk comorbidity (diabetes mellitus, moderate renal insufficiency, or LVEF ≤ 40%). Thakkar et al. identified adults with severe AS undergoing TAVR using administrative codes and stratified patients based on baseline SGLT2 inhibitor use, applying propensity‐score matching.

*Intervention (I)*: SGLT2 inhibitor therapy varied across studies. Paolisso et al. classified patients based on antidiabetic therapy at discharge, with SGLT2 inhibitor users receiving dapagliflozin or empagliflozin (doses not specified). Roubin et al. initiated dapagliflozin 10 mg once daily after TAVR. Thakkar et al. assessed pre‐TAVR SGLT2 inhibitor use, without specification of agents or dosing.

### 3.2 Risk of Bias (RoB) Assessment

We assessed the quality of each study based on its study design. The RCT (Roubin et al.) was assessed using the RoB 2 tool, which revealed an overall low risk of Bias (Figures S1 and S2).

The two observational studies, Paolisso et al. and Thakkar et al., were assessed using the NOS, which evaluates study quality across selection, comparability and outcome domains. Each study scored 8 out of 9, indicating high methodological quality and low risk of bias (Table S2).

## 4 Outcomes

### 4.1 Composite Outcome

**FIGURE 2:**
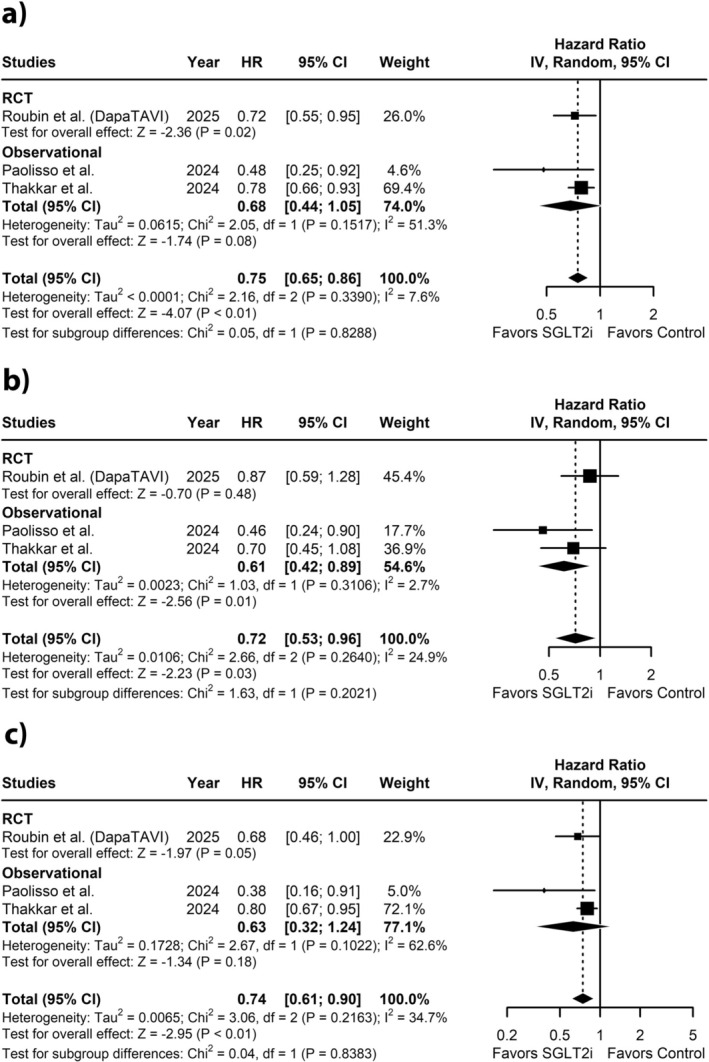
Forest plot for (a) Composite outcome (b) All‐cause mortality (c) HF hospitalisation.

**FIGURE 3:**
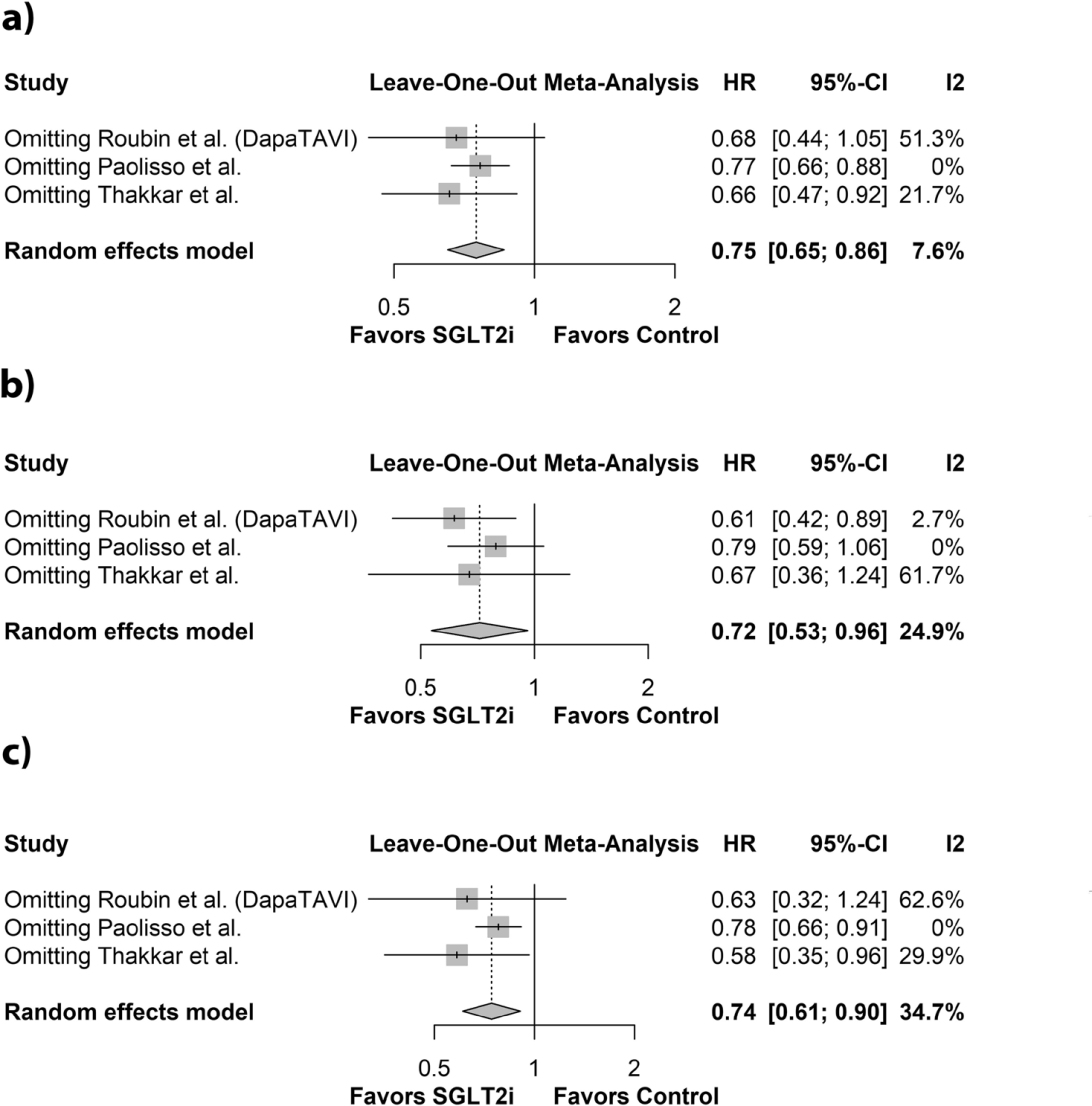
Leave one out sensitivity analysis for (a) Composite outcome (b) All‐cause mortality (c) HF hospitalisation.

To compare the composite outcome between the two groups, three studies including 3187 patients were pooled. The pooled analysis showed that the SGLT2 inhibitor treated group was associated with a significantly reduced composite outcome compared to the non‐SGLT2 inhibitor treated group (HR: 0.75; 95% CI: 0.65 to 0.86; *p* < 0.01; *I* ^2^ = 7.6%; Figure 2a). Sensitivity analysis excluding Paolisso et al. reduced the heterogeneity to 0%, without significantly altering the effect estimate (HR: 0.77; 95% CI: 0.66 to 0.88; Figure 3a). No effect modification was observed based on study design (*p* = 0.83; Figure 2a).

### 4.2 All‐Cause Mortality

A pooled analysis of 3 studies, comprising 3187 patients, was conducted to assess all‐cause mortality between SGLT2 inhibitor treated and non‐SGLT2 inhibitor treated groups. The analysis revealed a statistically significant reduction in all‐cause mortality in the SGLT2 inhibitor treated group (HR: 0.72; 95% CI: 0.53 to 0.96; *p* = 0.03; *I* ^2^ = 24.9%; Figure 2b). Excluding Paolisso et al. reduced heterogeneity to 0% while also producing a significant change in the effect estimate (HR: 0.79; 95% CI: 0.59 to 1.06; Figure 3b). No effect modification was observed based on study design (*p* = 0.20; Figure 2b).

### 4.3 HF Hospitalisations

Three studies, having a total of 3187 patients, assessed the impact of SGLT2 inhibitors on HF hospitalisations. The pooled analysis demonstrated a significant reduction in HF hospitalisations in the SGLT2 inhibitor treated group compared to the non‐SGLT2 inhibitor group (HR: 0.74; 95% CI: 0.61 to 0.90; *p* < 0.01; *I* ^2^ = 34.7%; Figure 2c). In the sensitivity analysis, excluding Paolisso et al. reduced heterogeneity to 0% and significantly altered the effect estimate (HR: 0.78; 95% CI: 0.66 to 0.91; Figure 3c). Effect modification by study design was not observed (*p* = 0.84; Figure 2c).

## 5 Discussion

In this meta‐analysis of 3187 patients with severe aortic stenosis undergoing TAVR, SGLT2 inhibitor therapy was associated with a significantly lower risk of the composite outcome of all‐cause mortality or heart failure hospitalisation at one year, as well as each component individually. To our knowledge, this is the first meta‐analysis to specifically evaluate the role of SGLT2 inhibitors in the post‐TAVR population, addressing an important evidence gap in contemporary valvular heart disease management.

Despite procedural success, heart failure readmissions remain common after TAVR and strongly predict subsequent mortality [22, 23]. This persistent risk reflects the fact that TAVR relieves valvular obstruction but does not reverse the chronic myocardial remodelling and fibrosis characteristic of advanced AS [24]. Our findings suggest that SGLT2 inhibitors, which are long established to reduce HF hospitalisations and mortality across a broad range of cardiac dysfunction [25, 26], may mitigate this residual vulnerability by targeting the underlying maladaptive myocardial substrate. The biological plausibility of this benefit is supported by evidence that SGLT2 inhibitors exert favourable effects on ventricular loading conditions, myocardial energetics and cardiac remodelling, as demonstrated in imaging and mechanistic studies [27, 28, 29, 30]. Furthermore, the renoprotective effects of SGLT2 inhibitors may provide additional value in mitigating post‐TAVR acute kidney injury, a common complication with significantly adverse prognostic implications [31, 32, 33].

Growing evidence also connects SGLT2 inhibition more directly to the pathobiology of aortic stenosis itself [11, 30, 34]. In a large retrospective cohort, Shah et al. reported that SGLT2 inhibitor use was associated with slower hemodynamic progression of AS [35]. Furthermore, the EASTER‐HF trial demonstrated improvements in LVEF and reductions in NT‐proBNP among patients receiving empagliflozin around the time of valve intervention [36]. Our analysis extends these observations by suggesting that physiological and mechanistic benefits may translate into tangible reductions in adverse clinical outcomes following TAVR.

## 6 Future Implications

The recent expansion of TAVR indications to include asymptomatic severe AS broadens its use to patients with less advanced myocardial disease [37]. This shift toward earlier intervention underscores the importance of adjunctive pharmacologic strategies capable of preventing or attenuating the progression of myocardial fibrosis. Preclinical studies demonstrating upregulated SGLT2 expression in calcified aortic valves, and its attenuation with empagliflozin treatment [30, 34], further support a potential disease‐modifying role for this drug class. As TAVR continues to move earlier in the disease continuum, therapies such as SGLT2 inhibitors may become increasingly relevant. Ongoing prospective trials, including the phase 4 ENAVO‐TAVR study evaluating enavogliflozin for the prevention of major cardiovascular events after TAVR [38], will be pivotal in determining the therapeutic role of SGLT2 inhibitors in post‐TAVR patients.

## 7 Limitations

Several limitations must be acknowledged. First, the majority of included studies were observational in nature. Although both observational studies provided adjusted effect estimates, the potential for residual confounding cannot be completely eliminated. Second, due to limited granularity in the reported data, we were unable to evaluate within‐class differences among SGLT2 inhibitors. Third, both the sensitivity analyses and subgroup analysis were based on an insufficient number of studies. Consequently, the overall pooled effect estimates were sensitive to the exclusion of single studies, which limits the reliability and interpretability of these findings. Finally, due to the small number of included studies, publication bias could not be formally assessed via funnel plots. In this emerging field, a high risk of publication bias remains, as unpublished negative or neutral findings may lead to an overestimation of the observed treatment effect.

## 8 Conclusion

In conclusion, this meta‐analysis provides preliminary signal that SGLT2 inhibitors may be associated with improved outcomes following TAVR. However, given the limitations of the current evidence, these findings must be considered exploratory. They underscore the need for dedicated, prospective randomised trials to definitively establish the efficacy and safety of SGLT2 inhibitors in this growing patient population. The ongoing phase 4 ENAVO‐TAVR trial [38] represents a critical step in this direction and may help clarify the role of SGLT2 inhibitors in the post‐TAVR treatment paradigm.

## Author Contributions

Conception and design of the work: S.M.D., A.K.; Data collection: N.M.K., I.G.; Data analysis: I.G., S.T., A.R.; Methodology: Z.A., A.R., M.B.; Drafting the article: H.T.G., S.L.F., S.A., S.M.D.; Critical revision of the article: All authors; Supervision: M.A.N., S.Z.A.; Validation: D.A.A., S.A; Funding Acquisition: None. All authors have significantly contributed to this paper and have approved the final version of the manuscript.

## Funding

The authors have nothing to report.

## Ethics Statement

Ethical approval was not required for this study, as it is a meta‐analysis using data from previously published research.

## Consent

The authors have nothing to report.

## Conflicts of Interest

The authors declare no conflicts of interest.

## Supporting information


**Data S1:** edm270184‐sup‐0001‐Supinfo.docx.
**Figure S1:** Traffic‐light plot showing RoB‐2 domain‐level judgements for included RCT.
**Figure S2:** Summary bar graph presenting overall RoB‐2 domain ratings for included RCT.
**Table S1:** Detailed search strategies used along with retrieved records.
**Table S2:** Newcastle–Ottawa Scale (NOS) Assessment Table of Included Cohort Studies.

## Data Availability

The data supporting the findings of this study are based on published literature. All relevant data extracted from these studies are available in the Supporting Information or can be made available upon reasonable request.
