# Supplementary material for: Efficacy of SGLT2 Inhibitors on Clinical Outcomes After Transcatheter Aortic Valve Replacement: A Systematic Review and Meta‐Analysis
Source: Endocrinol Diabetes Metab. 2026 Feb 26;9(2):e70184. doi: 10.1002/edm2.70184 (PMC12942055; doi:10.1002/edm2.70184)
Supplement: Supplementary file 1 — Data S1: edm270184‐sup‐0001‐Supinfo.docx. Figure S1: Traffic‐light plot showing RoB‐2 domain‐level judgements for included RCT. Figure S2: Summary bar graph presenting overall RoB‐2 domain ratings for included RCT. Table S1: Detailed search strategies used along with retrieved records. Table S2: Newcastle–Ottawa Scale (NOS) Assessment Table of Included Cohort Studies. [file EDM2-9-e70184-s001.docx]

**Supplementary File**

| **DATABASE/ SEARCH ENGINE** | **SEARCH STRING** | **RESULTS** |
| --- | --- | --- |
| PubMed | ("Transcatheter Aortic Valve Replacement" OR TAVR OR "Transcatheter Aortic Valve Implantation" OR TAVI OR "Catheter-Based Aortic Valve Replacement" OR "Percutaneous Aortic Valve Replacement" OR "Percutaneous Aortic Valve Implantation" OR "Transcatheter Aortic Valve Procedure" OR "Transfemoral Aortic Valve Replacement" OR "Transapical Aortic Valve Replacement" OR "Aortic Valve Implantation" OR "Minimally Invasive Aortic Valve Replacement") AND ("SGLT2 inhibitors" OR "SGLT-2 inhibitors" OR "Sodium-Glucose Cotransporter 2 Inhibitors" OR "Sodium-Glucose Co-Transporter 2 Inhibitors" OR "Sodium-Glucose Transporter 2 Inhibitors" OR "Glucose Cotransporter Inhibitors" OR "SGLT2 blockade" OR "SGLT2 inhibition" OR "Sodium-glucose transporter inhibitors" OR "SGLT inhibitor" OR "Gliflozin" OR "Dapagliflozin" OR "Empagliflozin" OR "Canagliflozin" OR "Ertugliflozin" OR "Ipragliflozin" OR "Remogliflozin etabonate") AND (Aortic Valve Stenosis [Mesh] OR Aortic stenosis OR Severe Aortic Stenosis OR "Symptomatic Aortic Stenosis" OR "Degenerative Aortic Stenosis" OR "Calcific Aortic Stenosis" OR "Senile Aortic Stenosis" OR "Aortic Valve Narrowing" OR "Aortic Valve Obstruction" OR "Left Ventricular Outflow Obstruction" OR "Aortic Valve Disease") | 10 |
| ScienceDirect | ("Transcatheter Aortic Valve Replacement") AND ("SGLT2 inhibitors") AND ("Aortic Stenosis") | 121 |
| Cochrane CENTRAL | ("Transcatheter Aortic Valve Replacement" OR TAVR OR "Transcatheter Aortic Valve Implantation" OR TAVI OR "Catheter-based Aortic Valve Replacement" OR "Aortic Valve Procedure" OR "Transcatheter Valve Procedure") AND ("SGLT2 inhibitor" OR "Sodium-Glucose Transporter 2 Inhibitors" OR "SGLT-2" OR gliflozin OR dapagliflozin OR empagliflozin OR canagliflozin OR ertugliflozin OR ipragliflozin OR luseogliflozin OR tofogliflozin OR "sodium-glucose" OR "glucose transporter inhibitor*" OR "type 2 diabetes drug*" OR "diabetes treatment") AND ("Aortic Stenosis" OR "Aortic Valve Stenosis" OR AS OR "Aortic Valve Disease") | 8 |
| Embase | ('transcatheter aortic valve replacement'/exp OR 'transcatheter aortic valve implantation'/exp OR 'aortic valve stenosis'/exp OR 'transcatheter aortic valve replacement':ti,ab OR TAVR:ti,ab OR 'transcatheter aortic valve implantation':ti,ab  OR TAVI:ti,ab OR 'catheter-based aortic valve replacement':ti,ab OR 'percutaneous aortic valve replacement':ti,ab OR 'percutaneous aortic valve implantation':ti,ab  OR 'transcatheter aortic valve procedure':ti,ab OR 'transfemoral aortic valve replacement':ti,ab OR 'transapical aortic valve replacement':ti,ab OR 'aortic valve implantation':ti,ab OR 'minimally invasive aortic valve replacement':ti,ab OR 'aortic stenosis':ti,ab OR 'severe aortic stenosis':ti,ab OR 'degenerative aortic stenosis':ti,ab  OR 'calcific aortic stenosis':ti,ab OR 'senile aortic stenosis':ti,ab OR 'aortic valve narrowing':ti,ab OR 'aortic valve obstruction':ti,ab OR 'left ventricular outflow obstruction':ti,ab OR 'aortic valve disease':ti,ab) AND ('sodium glucose transporter 2 inhibitor'/exp OR 'sglt2 inhibitor*':ti,ab OR 'sglt-2 inhibitor*':ti,ab OR 'sodium-glucose cotransporter 2 inhibitor*':ti,ab OR 'sodium-glucose co-transporter 2 inhibitor*':ti,ab OR 'glucose cotransporter inhibitor*':ti,ab OR 'sglt2 blockade':ti,ab  OR 'sglt2 inhibition':ti,ab OR 'sglt inhibitor*':ti,ab OR gliflozin:ti,ab OR dapagliflozin:ti,ab OR empagliflozin:ti,ab OR canagliflozin:ti,ab OR ertugliflozin:ti,ab OR ipragliflozin:ti,ab OR 'remogliflozin etabonate':ti,ab) | 110 |
| Scopus | (TITLE-ABS-KEY("transcatheter aortic valve replacement" OR TAVR OR "transcatheter aortic valve implantation" OR TAVI OR "catheter-based aortic valve replacement" OR "percutaneous aortic valve replacement" OR "percutaneous aortic valve implantation" OR "transcatheter aortic valve procedure" OR "transfemoral aortic valve replacement" OR "transapical aortic valve replacement" OR "aortic valve implantation" OR "minimally invasive aortic valve replacement" OR "aortic stenosis"  OR "severe aortic stenosis" OR "degenerative aortic stenosis" OR "calcific aortic stenosis" OR "senile aortic stenosis" OR "aortic valve narrowing" OR "aortic valve obstruction" OR "left ventricular outflow obstruction" OR "aortic valve disease"))  AND (TITLE-ABS-KEY("SGLT2 inhibitors" OR "SGLT-2 inhibitors" OR "sodium-glucose cotransporter 2 inhibitors" OR "sodium-glucose co-transporter 2 inhibitors"  OR "sodium-glucose transporter 2 inhibitors" OR "glucose cotransporter inhibitors"  OR "SGLT2 blockade" OR "SGLT2 inhibition" OR "SGLT inhibitor" OR gliflozin  OR dapagliflozin OR empagliflozin OR canagliflozin OR ertugliflozin  OR ipragliflozin OR "remogliflozin etabonate")) | 251 |

**Supplementary Table S1:** Detailed search strategies used along with retrieved records.

| **Criteria** | **Paolisso et al, 2024** | **Thakkar et al, 2024** |
| --- | --- | --- |
| **Selection (Maximum 4 points)** | | |
| (1) Representativeness of the exposed cohort | ★ | ★ |
| (2) Selection of the non-exposed cohort | ★ | ★ |
| (3) Ascertainment of exposure | ★ | ★ |
| (4) Demonstration that the outcome of interest was not present at the start of the study | ★ | ★ |
| **Comparability (Maximum 2 points)** | | |
| (5) Comparability of cohorts on the basis of the design or analysis | ★ | ★★ |
| **Outcome (Maximum 3 points)** | | |
| (6) Assessment of outcome | ★ | ★ |
| (7) Was the follow-up long enough for outcomes to occur | ★ | ★ |
| (8) Adequacy of follow-up of cohorts | ★ | - |
| **Total score** | 8 Stars | 8 Stars |

**Supplementary Table S2:** Newcastle–Ottawa Scale (NOS) Assessment Table of Included Cohort Studies.


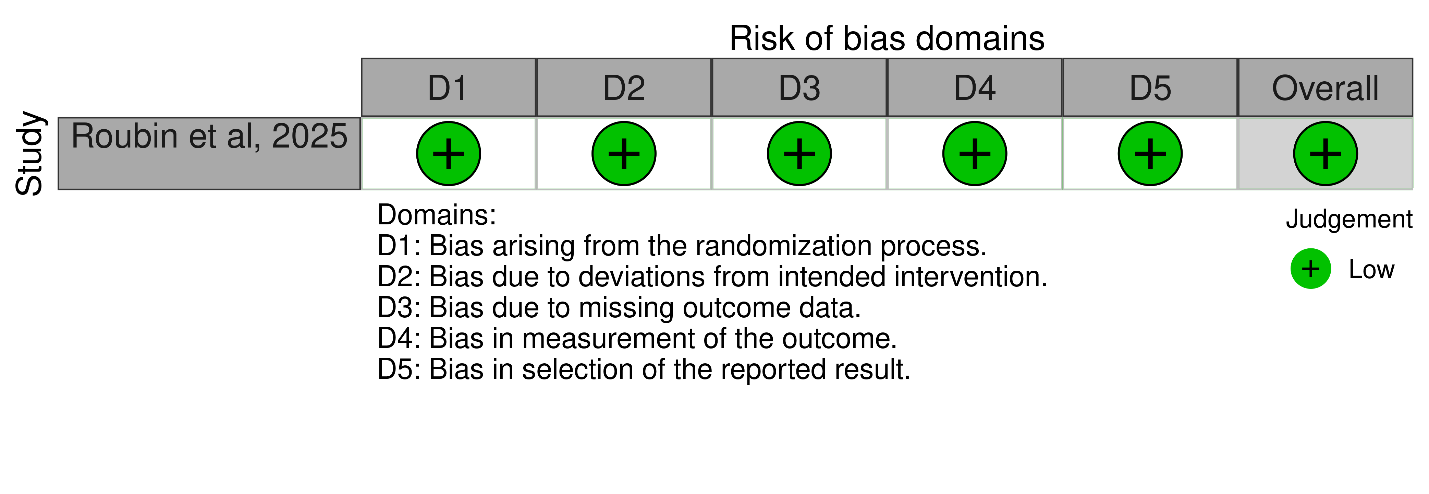


**Supplementary Figure S1:** Traffic-light plot showing RoB-2 domain-level judgments for included RCT.


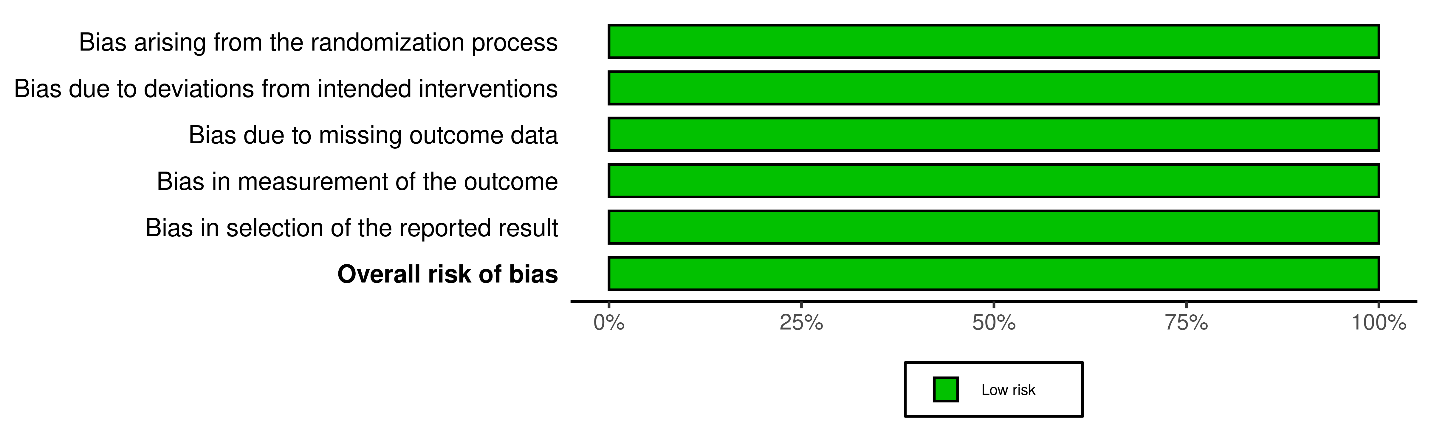


**Supplementary Figure S2:** Summary bar graph presenting overall RoB-2 domain ratings for included RCT.
